# Simulating the Hydrodynamic Conditions of the Human Ascending Colon: A Digital Twin of the Dynamic Colon Model

**DOI:** 10.3390/pharmaceutics14010184

**Published:** 2022-01-13

**Authors:** Michael Schütt, Connor O’Farrell, Konstantinos Stamatopoulos, Caroline L. Hoad, Luca Marciani, Sarah Sulaiman, Mark J. H. Simmons, Hannah K. Batchelor, Alessio Alexiadis

**Affiliations:** 1 School of Chemical Engineering, University of Birmingham, Edgbaston, Birmingham B15 2TT, UK; konstantinos.x.stamatopoulos@gsk.com (K.S.); M.J.Simmons@bham.ac.uk (M.J.H.S.); 2 Biopharmaceutics, Pharmaceutical Development, PDS, MST, RD Platform Technology & Science, GSK, David Jack Centre, Park Road, Ware, Hertfordshire SG12 0DP, UK; 3 Nottingham Digestive Diseases Centre and National Institute for Health Research (NIHR) Nottingham Biomedical Research Centre, Nottingham University Hospitals NHS Trust and University of Nottingham, Nottingham NG7 2UK, UK; Caroline.L.Hoad@nottingham.ac.uk (C.L.H.); Luca.Marciani@nottingham.ac.uk (L.M.); Sarah.Sulaiman@nottingham.ac.uk (S.S.); 4 Sir Peter Mansfield Imaging Centre, School of Physics and Astronomy, University of Nottingham, Nottingham NG7 2RD, UK; 5 Strathclyde Institute of Pharmacy and Biomedical Sciences, University of Strathclyde, 161 Cathedral Street, Glasgow G4 0RE, UK; Hannah.Batchelor@strath.ac.uk

**Keywords:** Dynamic Colon Model (DCM), digital twin, discrete multiphysics, Smoothed Particle Hydrodynamics (SPH), large intestine, colon, shear rate, dissolution apparatus, Magnetic Resonance Imaging (MRI), colon targeted drug delivery

## Abstract

The performance of solid oral dosage forms targeting the colon is typically evaluated using standardised pharmacopeial dissolution apparatuses. However, these fail to replicate colonic hydrodynamics. This study develops a digital twin of the Dynamic Colon Model; a physiologically representative in vitro model of the human proximal colon. Magnetic resonance imaging of the Dynamic Colon Model verified that the digital twin robustly replicated flow patterns under different physiological conditions (media viscosity, volume, and peristaltic wave speed). During local contractile activity, antegrade flows of 0.06–0.78 cm s^−1^ and backflows of −2.16–−0.21 cm s^−1^ were measured. Mean wall shear rates were strongly time and viscosity dependent although peaks were measured between 3.05–10.12 s^−1^ and 5.11–20.34 s^−1^ in the Dynamic Colon Model and its digital twin respectively, comparable to previous estimates of the USPII with paddle speeds of 25 and 50 rpm. It is recommended that viscosity and shear rates are considered when designing future dissolution test methodologies for colon-targeted formulations. In the USPII, paddle speeds >50 rpm may not recreate physiologically relevant shear rates. These findings demonstrate how the combination of biorelevant in vitro and in silico models can provide new insights for dissolution testing beyond established pharmacopeial methods.

## 1. Introduction

In recent years, colon-targeted drug delivery has received increased attention due to regional conditions that present advantages for the delivery of certain types of pharmaceutical formulation compared to the small intestine [1,2]. The hydrodynamics of the proximal colon are crucial for the design and optimisation of colon-targeted formulations, particularly in terms of disintegration, dissolution, and distribution of the dosage form. To gain a better understanding of the hydrodynamics and mixing conditions in the intestinal environment, in vitro, as well as in silico, studies have been carried out, focusing on both the colon [3,4,5,6,7] and the small intestine [8,9].

**Figure 1:**
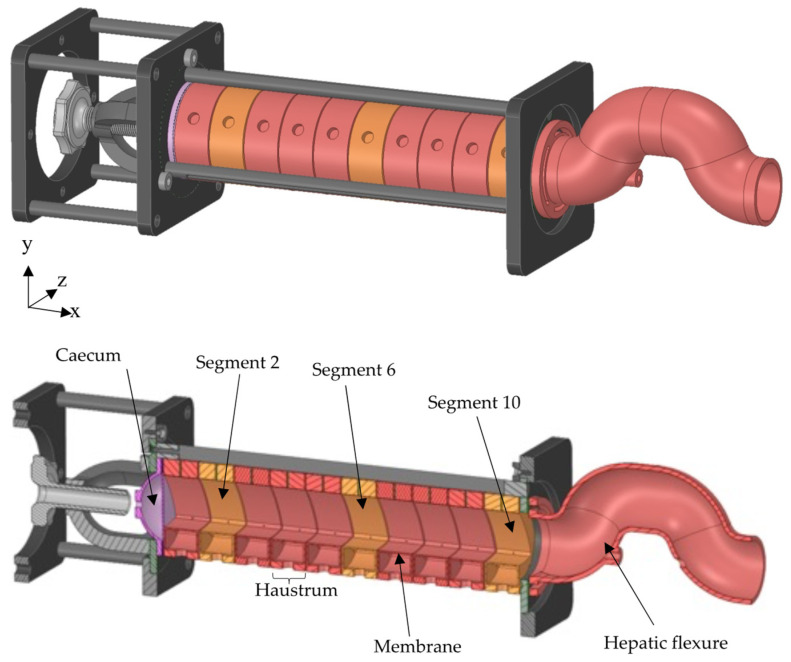
Schematic of the Dynamic Colon Model (DCM), Adapted from [12], MDPI, 2021. The DCM has a segmented appearance reflecting that of the human proximal colon: segment 1 is adjacent to the *caecum*, through to segment 10 adjacent to the *hepatic flexure*.

In Vitro dissolution apparatuses have historically been used for biopredictive testing. Although pharmacopeial dissolution apparatuses permit the control of media properties, the vessels bear little semblance to colonic geometry and use simplified mixing methods that fail to reproduce the hydrodynamic conditions of the human colon in vivo [10,11]. The Dynamic Colon Model (DCM), depicted in Figure 1, is a biorelevant in vitro model that replicates the architecture of the proximal colon and reproduces peristaltic/segmental activity [6,7].

The design of the DCM was based on clinical data obtained from MRI images of the human (adult) proximal colon in vivo. The DCM is able to mimic the motor patterns of the colon, which mostly occur as propagating pressure waves (PPWs): one of the identified motor patterns in the colon [13]. The DCM is the most physiologically relevant in vitro colon model to date as it is the only model that replicates peristaltic motility in a lumen with the segmented architecture of the human colon [14]. A recent study has shown that when a PPW is applied to the DCM, the motion of the walls causes the contents of the lumen to flow in a way that closely reproduces the flow in the human proximal colon [12,15], verifying the hydrodynamics of the model. 

In Vitro and in silico models that are based on in vivo data offer affordable alternatives to in vivo studies. Furthermore, in vivo studies are conducted, where possible, using healthy volunteers, and this population does not represent the extremes of GI variability which are of interest in the design of a dosage form. The DCM can reproducibly replicate extreme GI motion. More advanced in vitro models that are physiologically representative offer the possibility of a deeper insight into in vivo conditions and therefore better understanding of the physical laws governing colonic space. This is especially important for pharmaceutical research and the development of new formulations of modified release solid oral dosage forms that reach the colon, as these data are necessary to predict release behaviour in the colonic environment. 

Over the last few years, several in silico models of the human proximal colon have been developed [3,5,16] based on a computational technique called Discrete Multiphysics (DMP) [17,18]. Recently, this approach has been applied to the pharmaceutical field and used to model drug release from a solid dosage form under the influence of different in vivo motility patterns [4]. The major advantage of in silico models is that they are resource-saving compared to in vitro models and especially to in vivo experiments. Additionally, in silico models are highly versatile and provide additional insights that are difficult to acquire using common measurement techniques, often at resolutions that are equally unattainable. However, in vitro models are essential to make sure all relevant variables occurring in the real environment are accounted for, and to generate sufficient data to inform the development and the validation of their digital counterpart. Therefore, the quality and quantity of the data describing the colonic environment will always depend on the power of in vitro and in silico models. Together, myriad runs can be conducted, generating a high data output at low cost. This data is crucial for the pharmaceutical industry to create effective therapeutic delivery vehicles.

**Figure 2:**
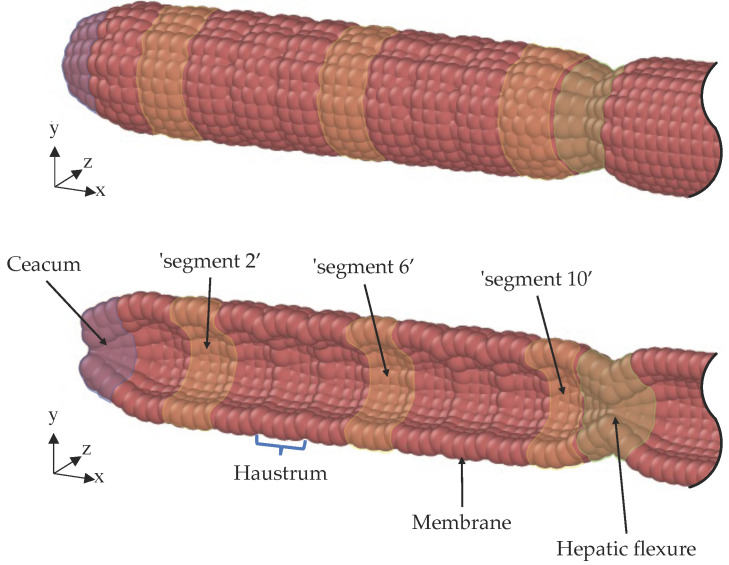
Schematic view (**top**) and a cross-sectional view (**bottom**) of the computational model (DCMDT). The DCMDT comprises 10 sections reflecting the DCM. Segment 1 is adjacent to the *caecum* and segment 10 is adjacent to the *hepatic flexure*. The *hepatic flexure* is modelled as a reduction to create a backpressure, guided by the in vivo situation.

This study describes the development and validation of a digital twin (DT) of the DCM (DCMDT) using a particle modelling approach. The DCMDT is depicted in Figure 2.

The DCMDT is a digital informational construct of the physical DCM that exists in virtual space. It replicates the design and motility of the DCM and is similarly compatible with a range of fluids, which is achieved by modifying the physical properties of the computational fluid particles. Further details on the modelling methodology are given in Section 2.2.

The environmental conditions inside the lumen of the proximal colon are controlled by a range of factors, including but not limited to disease state, microbiota, prandial state, ingested food contents, and importantly, the inherent interindividual variation [19]. The dynamic interplay of these influences can affect a wide range of parameters, which can ultimately be manipulated in the in vitro or/and in silico models. For example, media volume can change with prandial state and could affect the sink conditions of a formulation, resulting in accelerated or hampered release of the active pharmaceutical ingredient (API) which can influence bioavailability [1,9]. Functional gastrointestinal disorders may affect the motility of the colonic walls; dampened motility may cause lower shear rates to be exerted on the surface of the dosage form, leading to incomplete release of the API. Contents of solid or liquid food ingested may affect the viscosity of the contents of the proximal colonic lumen [19]. A more viscous fluid demonstrates greater resistance to flow and may cause a different velocity profile in the lumen, affecting the transport and shear forces acting on a dosage form [6,7].

The DCM and its DT permit the manipulation of these parameters individually, under fixed conditions, to scrutinise the effects. Thus, this study investigates how the interplay of media viscosity, media volume, and wall motility influence flows inside the DCM (Figure 1) and the DCMDT (Figure 2). This will facilitate assessment of the ability of the DCMDT to replicate the wall motion and the relationship this has with the flow of the contents. Flow analysis will cover the velocity and shear rate distributions at different locations along the models. Shear rates within the fluid determine the shear stresses exerted by the fluid on the surface of a dosage form in the colonic lumen, which governs the erosion of solid oral dosage forms inside the colon [4]. The ability of the DCMDT to extract shear rate data under a multitude of conditions with relative ease could establish it as a highly valuable tool to inform the design of formulations that are sensitive or insensitive to motion.

## 2. Methodology

### 2.1. Experimental Work

Experimentally, a simulated antegrade PPW travelling from the *caecum* to the *hepatic flexure* was applied to the DCM and the velocity of the contents and the shear rate in the lower layer of fluid closest to the bottom wall were measured. The study investigated the effects of three factors: propagation speed of the contractile wall wave, media viscosity, and volume on the results as a full factorial design. In vitro measurements were made using phase contrast (PC) cine-MRI.

In the DCM, volume was varied from 150 to 200 mL, corresponding to filling levels of approximately 60% and 80% respectively. Viscosity was controlled by varying aqueous sodium carboxymethyl cellulose (NaCMC) concentration. The low viscosity fluid (LOVIS) consisted of 0.25% (*w*/*v*) NaCMC aqueous solution whilst the high viscosity fluid (HIVIS) was a 0.50% (*w*/*v*) NaCMC aqueous solution. Details of the fluids used are given in Section 2.2.2.2. The motility pattern was varied by controlling the speed of the propagating wave along the DCM wall, varied between 0.4 and 0.8 cm s^−1^. The occlusion degree was fixed at 60 ± 5% for each pattern.

#### 2.1.1. MRI Protocol

Scanning was carried out using a 3T Philips Ingenia widebore scanner (Philips, Best, The Netherlands). Localiser scans were carried out prior to the tagging and PC scans for placement of these sequences across the DCM.

PC scans were conducted using a sequence adapted from a standard PC flow sequence that usually acquires multiple flow measurements in blood vessels throughout the cardiac cycle, described in detail in [20]. In this work, a single fast field echo (FFE) image of 101 × 101 voxels was generated using flow-sensitive gradients. The scan was repeated for each parameter combination investigated. The parameters from the MRI scanner are shown in Table 1.

Three different slice locations along the length of the DCM were used to investigate the spatial variation of the flow induced; at segment 2, close to the mimic *caecum*, segment 6, midpoint and segment 10, *hepatic flexure* (see Figure 1) sequentially with 10 s rest periods between scans. Following completion of all spatial locations for the default motility pattern, the protocol was repeated for the slower PPW. After completion of all scans, media volume and/or media type (LOVIS or HIVIS) were changed, and the protocol repeated. The flow was encoded only in the streamwise direction (*x*-axis). Maximum velocities were encoded at ±3 cm s^−1^ based on previous work by O’Farrell et al. [12]. Positive and negative velocities represent flow along the x-axis towards the *hepatic flexure* and *caecum* (depicted in Figure 1) respectively.

To account for the background signal, initial velocity measurements were taken using PC cine-MRI prior to any induced motility (neutral wall position) when it was known the luminal contents were at rest. The mean velocity over the cross-sectional lumen flow area was close to zero at 4.32 × 10^−4^ cm s^−1^ with a standard deviation of 6.40 × 10^−3^ cm s^−1^. This standard deviation value was taken as the measurement error for a single voxel and hence accumulates in the error for PC cine-MRI mean velocity measurements.

### 2.2. Modelling Approach

**Figure 3:**
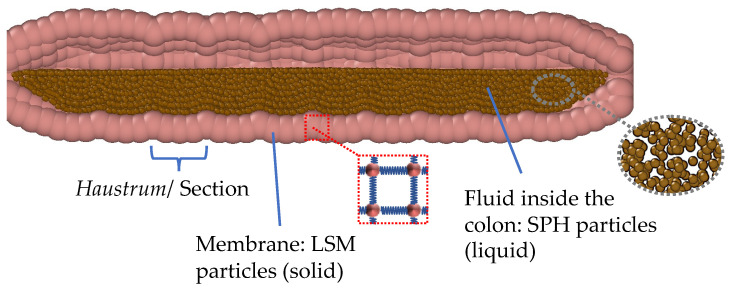
Particle representation of the model showing the colon haustra, the flexible membrane, and the fluid inside the colon.

The DCMDT employs Discrete Multiphysics (DMP), similar to Schütt et al. [5]. DMP is a meshless particle-based simulation technique where computational particles are used instead of a computational grid. DMP couples different particle-based modelling techniques, such as Smoothed Particle Hydrodynamics (SPH) Lattice Spring Model (LSM), and Discrete Element Method (DEM). The model in this study only accounts for SPH and LSM. SPH is used to model the fluid by calculating the viscous and pressure forces between the particles that represent the fluid. LSM is used to calculate the elastic forces between the particles that represent the solid walls of the DCM. The particle types and details of the model are highlighted in the cross section of the partially filled DCMDT in Figure 3. This partially filled state reflects the average situation where gas is also present in the colon.

Further details on the DT and the simulation parameters are given in Section 2.2.2. For a general overview on the DMP theory and how it can be applied to a variety of applications such as biological flows and/or fluid–structure interactions [3,5,21,22,23,24,25,26,27], solidification and dissolution [28,29,30], machine learning [31,32], and composite materials [33], the reader can refer to the available literature (e.g., DMP: [17,18], SPH: [34], LSM: [35,36,37]). For technical details and how it is applied to the large intestine, the reader is referred to Refs. [3,5,16].

#### 2.2.1. DCMDT Geometric Design

**Figure 4:**
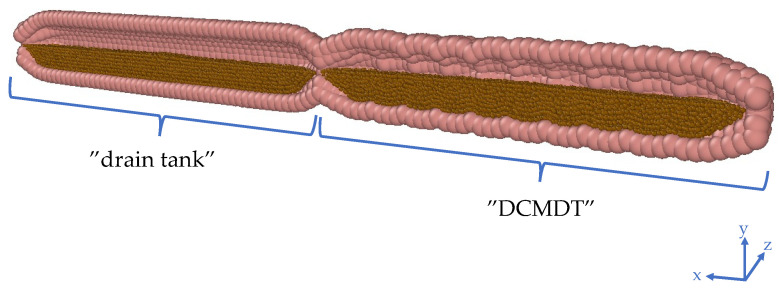
Cross section of the digital twin of the DCM and the ‘drain tank’. The antegrade direction in this image is from the right to the left.

The DCMDT replicates the geometry and segmental appearance of the DCM, which is a biorelevant model of the human proximal colon (see Figure 1) [6,7]. It is composed of a cylindrical body with a total length of 0.622 m and an inner diameter of 4.0 × 10^−2^ m. Only 0.24 m of the total model represents the DCM whereas the remaining part serves as a ‘drain tank’ (Figure 4).

In the DCM, an antegrade PPW propels the fluid towards a rigid siphon that represents the *hepatic flexure* at the end of the DCM (see Figure 1); the sharp bend between the proximal and the transverse colon. Here, the fluid rises up the rigid siphon and falls back down when the PPW ends and the haustra return to the neutral position. The DCMDT is a closed system that mimics the presence of the *hepatic flexure* by separating the DCM-like compartment from the drain tank by constriction, enabling a small portion of fluid to escape the DCMDT lumen, if necessary, whilst still generating a back pressure when the wave reaches the end of the lumen.

**Figure 5:**
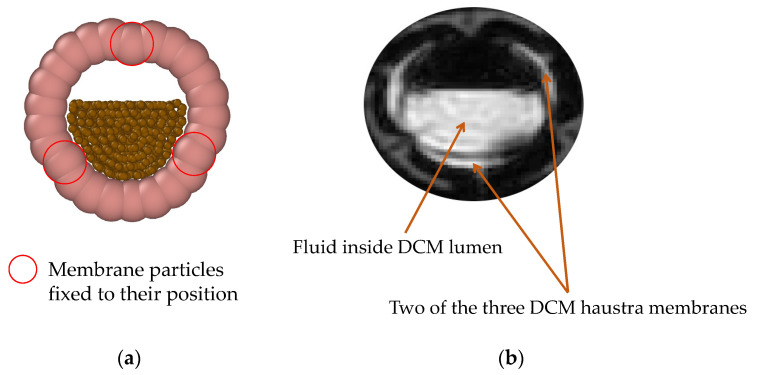
Shape of the segment during relaxing, where (**a**) is the computational model and (**b**) is a segment of the DCM.

The DCM consists of 10 individual segments of equal size. Each segment consists of three chambers, representing the sack-like haustra on the human colon, which are controlled simultaneously to contract and relax the wall for each segment. In the DCMDT, the membrane is also divided into 10 segments of equal size. Each segment consists of 3 circular rings of 25 LSM ‘wall’ particles, one of which can be seen in Figure 5a.

To mimic the shape of the DCM segments during the relaxation and contraction phases, three particle rows along the DCMDT are fixed in position as highlighted in Figure 5a. This prevents them from moving during relaxation or contraction and consequently creates a similar three chamber system.

Membrane motion is segmental in that the rings inside each segment move together as one body through the radial axis, contracting and relaxing in response to the application of a positive or negative radial force and mimicking contraction and relaxation of the DCM membrane respectively. The radial motion of adjacent segments can be synchronised to replicate any DCM motility pattern in terms of contraction/relaxation pattern, luminal occlusion degree, and the speed that a contractile wave propagates along the colonic axis.

#### 2.2.2. DCMDT and Computational Simulation Parameters

##### 2.2.2.1. Membrane Design and Motility

The membrane is modelled similarly to [5]. The DCMDT membrane is represented by 975 LSM particles in total which are tethered to their initial position using a Hookean spring, so that the membrane particles return to their initial position after the activation by a radial force (i.e., contraction or relaxation). This also fixes the model in the domain during the simulation. Additionally, particles in close proximity are interconnected with an additional Hookean force. Analogously, the forces are calculated using Hooke’s law:

(1) $$F_{ij}=k\left(r_{ij}-r_{0}\right),$$

where *F_ij_* represents the present spring force between particle *i* and *j* and *k* is the Hookean constant. The current distance between the particles *i* and *j* is represented by *r_ij_*, while *r_0_* is the equilibrium distance between these particles. This creates a lattice structure that replicates the properties of an elastic solid [35]. This approach has been used previously to model biological membranes [25,38]. The Hookean coefficient used for the lattice is *k_M,b_*, the coefficient used for the tethered springs is *k_M,p_*. An additional viscous force

(2) $$F_{i}=-k_{M,v}v_{i},$$

where *v_i_* is the velocity of the particle, is added to the membrane particles to improve the stability of the simulation and simultaneously confer viscoelastic properties to the membrane as in [39].

Once the forces acting on each particle are calculated, the particles move according to the Newton equation of motion

(3) $$m_{i}\frac{dr_{i}}{dt}=\overset{N}{\underset{j}{\sum }}F_{ij},$$

where **r***_i_* is the position of particle *i*. The pattern of force application to the simulated wall follows that of the DCM, wherein the rate of relaxation from peak contraction to neutral position is slower than the rates of initial relaxation and contraction. This is intended to mimic the viscoelasticity of the intestinal wall in vivo. Further details of the simulated membrane are shown in Table 2.

##### 2.2.2.2. Fluid

Two different fluid volumes of 150 and 200 mL (i.e., 60% and 80% respectively) were modelled with SPH particles. A resolution analysis to determine the number of SPH particles representing the fluid was carried out in [5]. The model also accounts for two different fluid viscosities, a LOVIS and a HIVIS fluid. The aqueous NaCMC solutions (see Section 2.1) used in the DCM lumen demonstrated a response to shear that follows the power law model (R^2^ = 0.999). Therefore, the shear stress *τ* can be calculated according to Equation (4):

(4) $$\tau =Κ{\dot{\gamma }}^{n},$$

where *K* is the consistency index, $\dot{\gamma }$ the shear rate and *n* the power law exponent. The parameters describing the fluids used are provided in Table 3.

**Figure 6:**
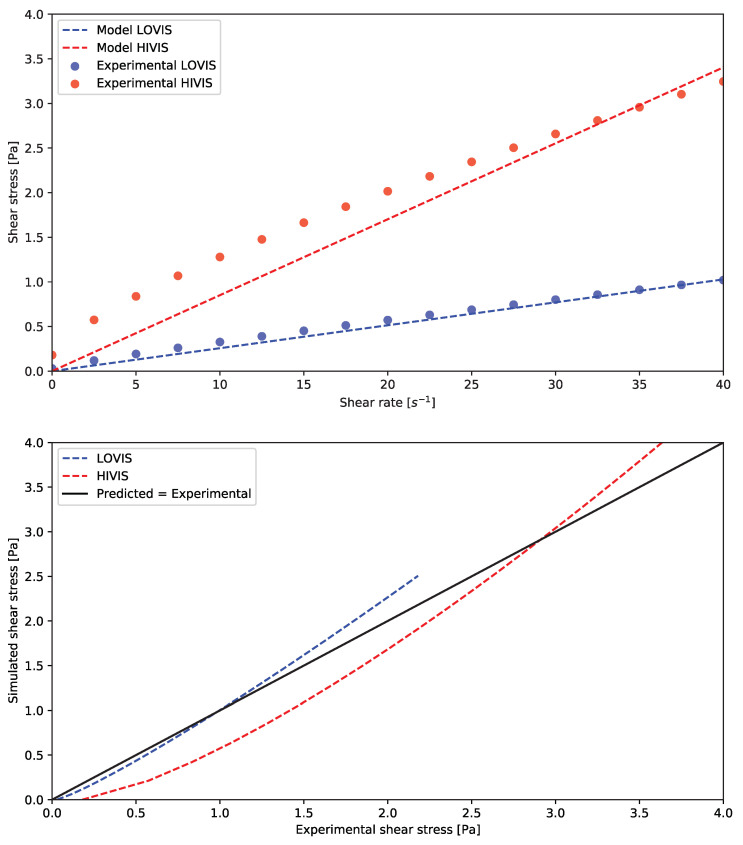
Rheological behaviour of LOVIS and HIVIS fluids in the DCM and the simulated counterparts in silico. Rheological measurements were carried out at 25 °C.

Figure 6 shows how the rheology of the simulated HIVIS and LOVIS fluids compares to the power law model fitted to the experimental data.

An approximately linear viscoelastic region was identified between 0–40 s^−1^ corresponding to a constant viscosity. Therefore, the fluid modelled in the DCMDT was assumed to be Newtonian for simplicity, with a viscosity equal to the gradient of the linear viscoelastic region; 26 mPa s (R^2^ = 0.9959) for the model LOVIS and 85 mPa s (R^2^ = 0.9806) for the model HIVIS fluid.

##### 2.2.2.3. Fluid Structure and Global Boundary Conditions

In the SPH framework the continuum domain is discretised into a finite number of points which can be thought of as particles, which are characterised by their mass, velocity pressure, and density. The SPH equations of motion result from the discrete approximations of the Navier–Stokes equation. SPH is based on the mathematical identity:

(5) $$f\left(r\right)=\int \int \int \;f\left(r’\right)\delta \left(r-r’\right)dr^{\prime },$$

where *f*(**r**) is any scalar function defined over the volume *V*. The vector **r** is position vector defined in the space *V*. *δ*(**r**) is the three-dimensional delta function and approximated in the SPH formulations by a smoothing kernel *W* and its characteristic width or smoothing length *h*:

(6) $$\underset{h\to 0}{\lim}W\left(r,h\right)=\delta \left(r\right)$$

A variety of kernel functions can be found in literature. In this study, the so-called Lucy kernel function [40] is used. By replacing the delta function by a kernel or smoothing function *W*, Equation (5) becomes

(7) $$f\left(r\right)\approx {\int \int \int }^{\;}f\left(r^{\prime }\right)W\left(r-r^{\prime },h\right)dr^{\prime }\;\;\;.$$

The discretisation over a series of particles of mass *m* = *ρ*(**r**’)*d***r**’, the identity equation results in

(8) $$f\left(r\right)\approx \underset{i}{\sum }\frac{m_{i}}{\rho_{i}}f\left(r_{i}\right)W\left(r-r_{i},h\right),$$

here, *m_i_* is the mass and *ρ_i_* is the density of *i*th particle, where *i* ranges over all particles within the smoothing kernel *W* $\left(i.e.,\;\left|r-r_{i}\right|<h\right)$ . Equation (8) represents the discrete approximation of a generic continuous field and can be used to approximate the Navier–Stokes equation

(9) $$m_{i}\frac{dv_{i}}{dt}=\underset{j}{\sum }m_{i}m_{j}\left(\frac{P_{i}}{\rho_{i}^{2}}+\frac{P_{j}}{\rho_{j}^{2}}+\prod_{i,j}\right)\nabla_{j}W_{i,j}+F_{i},$$

where vi is the velocity of particle *i*, *P* is the pressure, *W_i,j_* is the concise form of *W*(**r***_j_*–**r***_i_*, *h*), the term ∇*_j_* is the gradient of the kernel with respect to the coordinate **r***_j_*. **F***_i_*, accounts for a body force (e.g., gravity) and Π*_i,j_* denotes the viscous forces. For the tensor Π*_i,j_*, there are different expressions available in the literature; here we use [41]

(10) $$\Pi_{i,j}=-\alpha h\frac{c_{0}}{\rho_{ij}}\;\frac{v_{ij\;}r_{ij}}{\rho_{ij}^{2}+b\;h^{2}}\;\;,$$

where *α* and *b* are dimensionless parameters to ensure the stability of the simulation. *c*_0_ is the reference speed of sound at zero applied stress and *v_ij_* represents the relative velocity and *ρ_ij_* is the density of particle *i* and *j*, respectively. The constant *b* is used with *b* ≈ 0.01. With the following relation, the artificial viscosity can be recognised as an effective kinematic viscosity *ν*. The value of *α* is chosen depending on the desired effective kinematic viscosity in the simulation, accordingly [42]:

(11) $$\nu =\frac{\alpha \;h\;c_{0}}{10}$$

To calculate the pressure forces between the fluid particles the Tait equation is used. This equation is also used to link the density *ρ* and the pressure *P* and correspondingly fulfil Equation (9):

(12) $$P=\frac{c_{0}{}^{2}\;\rho_{0}}{7}\left[{\left(\frac{\rho }{\rho_{0}}\right)}^{7}-1\right]\;\;\;.$$

Here, *ρ*_0_ the reference density at zero applied stress. Further details of the fluid properties are shown in Table 4.

To imitate the solid–fluid interactions (i.e., between the wall and the boundary layer of luminal fluid) a repulsive potential is used. This potential is used for the purpose of avoiding overlap between solid and liquid particles. A soft potential of the following form is used:

(13) $$E_{ij}=A\left[1+cos\left(\frac{\pi \;r_{ij}}{r_{c}}\right)\right]\;\;\;\;\;\;\mathrm{with}\;\;\;\;\;\;r_{ij}<r_{c},$$

where *A* is an energy constant, *r_ij_* represents the distance between particle *i* and *j* and *r_c_* is the cut-off distance. The no-slip boundary conditions between the solid and fluid particles are approximated by viscous forces similar to those of Equation (10), but applied to the interaction between the solid and the fluid particles.

Model parameters of the DCMDT used in the simulations are presented in Table 5:

### 2.3. Software

The computational simulations in this study were performed using the University of Birmingham BlueBEAR HPC service [43], running the simulations on 10 cores with 40 GB of memory, resulting in a simulation time of about 10 min each. The open-source code LAMMPS [44,45] is used for the numerical calculations and the open-source code OVITO [46] for the visualisation of the results from the computational simulations. MATLAB [47] is used for the visualisation of the experimental data and the postprocessing of the DCMDT data as well as the experimental data.

### 2.4. Method of Analysis

#### 2.4.1. MRI Data Analysis

**Figure 7:**
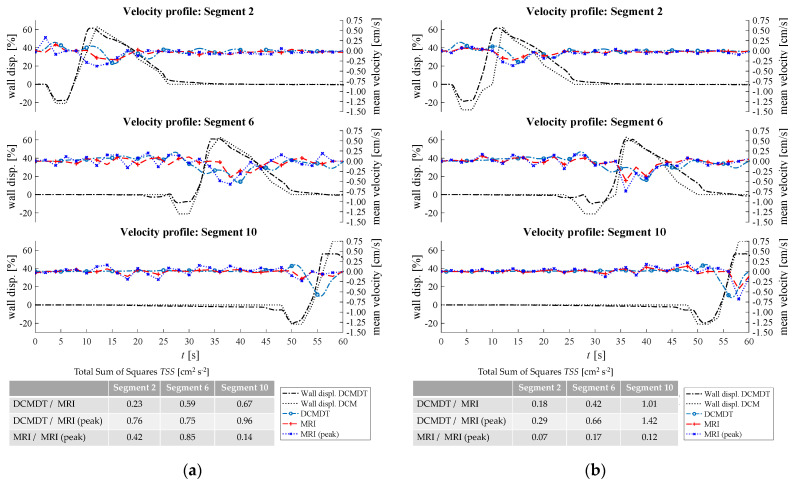
Comparison of the fluid velocities and wall displacement profiles of the DCM and the DCMDT with 60 % fluid volume and slower propagating PPW. Parts (**a**,**b**) compare the mean fluid velocities with LOVIS and HIVIS respectively.

**Figure 8:**
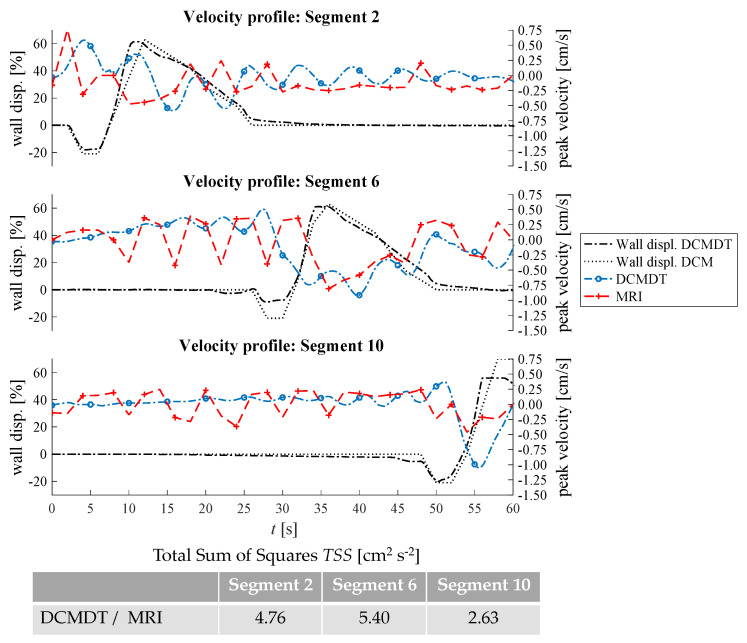
Comparison of the maximum fluid velocities and wall displacement profiles of the DCM and the DCMDT at low fluid volume, low fluid viscosity, and slow propagating PPW conditions are compared.

**Figure 9:**
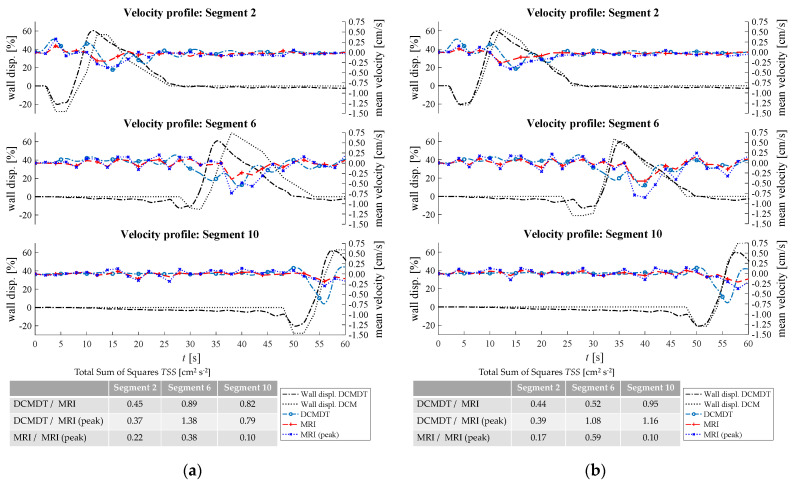
Comparison of the fluid velocities and wall displacement profiles of the DCM and the computational model at high fluid volume and slow propagating PPW. In (**a**) the mean fluid velocities at low fluid viscosity and in (**b**) the mean fluid velocities at high fluid viscosity are compared.

**Figure 10:**
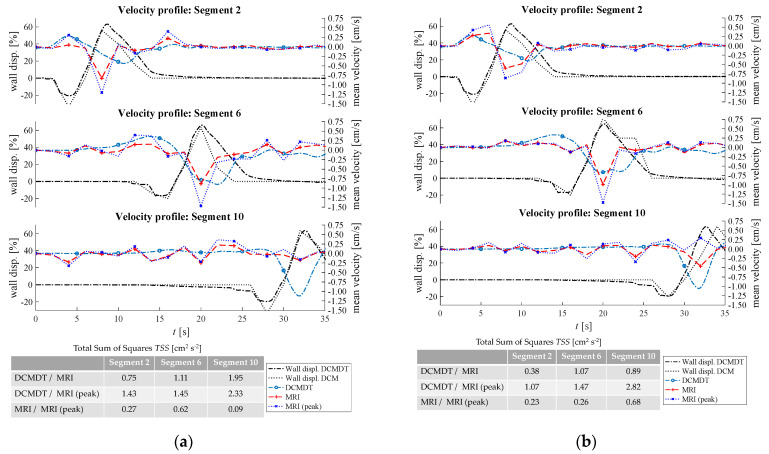
Comparison of the fluid velocities and wall displacement profiles of the DCM and the DCMDT at low fluid volume and fast propagating PPW. In (**a**) the mean fluid velocities at low fluid viscosity and in (**b**) the mean fluid velocities at high fluid viscosity are compared.

**Figure 11:**
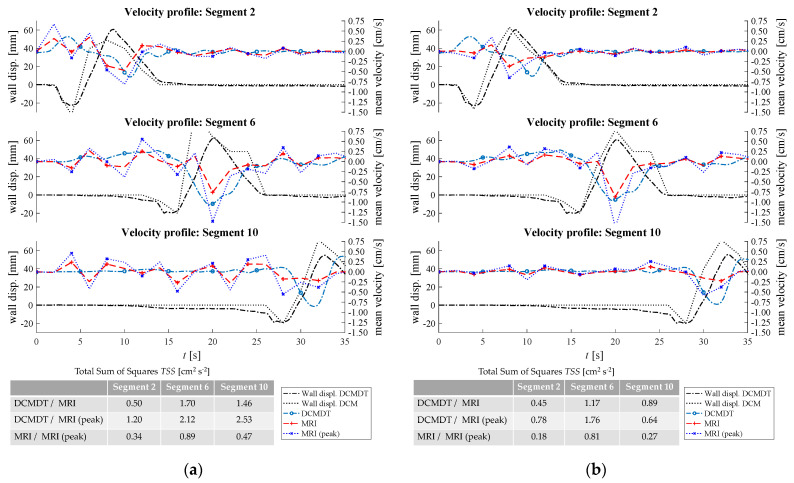
Comparison of the fluid velocities and wall displacement profiles of the DCM and the DCMDT at high fluid volume and fast propagating PPW. In (**a**) the mean fluid velocities at low fluid viscosity and in (**b**) the mean fluid velocities at high fluid viscosity are compared.

**Figure 12:**
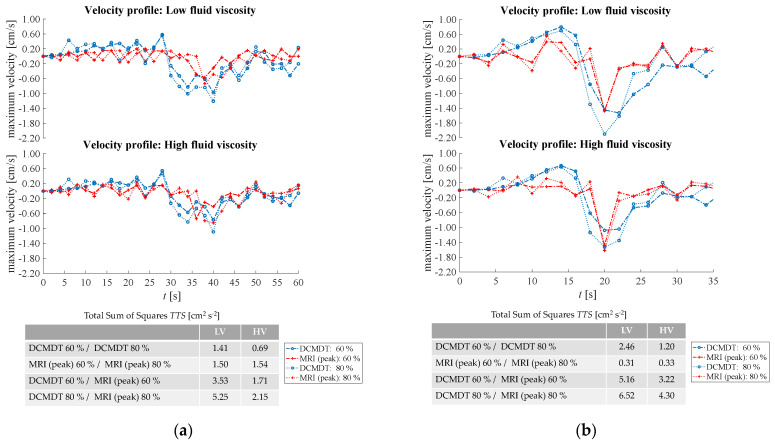
Comparison of the fluid velocities at different fluid volumes and different fluid viscosities of segment 6. (**a**) represents data for the slower propagating PPW and (**b**) for the faster propagating PPW (**b**). In the table for the Total Sum of Squares, the following abbreviations are used: LV—low viscosity, HV—high viscosity.

Using PC cine-MRI, the mean velocity of the DCM lumen contents was measured by taking the mean of all weighted-average velocities measured in voxels that constitute the through-plane lumen cross sectional flow area (denoted as ‘MRI’ in Figure 7, Figure 8, Figure 9, Figure 10, Figure 11 and Figure 12). Additionally, peak velocity was estimated by taking the mean of the five voxels in the centre of the lumen (denoted as ‘MRI (peak)’ in Figure 7, Figure 10, Figure 11 and Figure 12), to assess the impact of any stagnant regions of fluid close to the walls on through-plane mean velocity. Furthermore, peak velocities were also measured by taking the mean of the four highest value pixels within each region of interest (ROI). Due to the potential for high noise in individual pixel velocity measurements, MRI peak velocity estimates should be made using several pixels, rather than just one [48]. The standard deviation of the mean velocity calculated using each ROI was considered to be the error associated with the MRI mean velocity measurement.

Since velocity was encoded only in the streamwise direction, *x*, as this is the principal direction of flow and it was assumed that the *z* and *y* components of velocity were of negligible magnitude. $v_{⊥i}$ is the measured streamwise component of velocity of the fluid in pixel *i*. The measured value represents the weighted average of streamwise velocity inside the area entrapped within the pixel, which is dictated by the spatial resolution of the scanner. The flow rate through the pixel can therefore be determined by the following equation where $q_{i}$ is flow rate through pixel *i*, and $a_{i}$ is the area of pixel *i*.

(14) $$q_{i}=v_{⊥i}a_{i}$$

The shear rate distribution can be mapped by evaluating the spatial gradient of the velocity distribution. Encoding velocity only in the streamwise direction simplifies the problem, eliminating the components of the shear rate tensor that involve measured velocity of the element of fluid inside pixel *i* in the *z*-direction, $v_{⊥z,i}$ and in the *y*-direction, $v_{⊥y,\;i}$ . Additionally, the gradient of streamwise velocity with respect to the change in *x*-direction becomes unattainable as velocity values in only a single slice are obtained, therefore $\frac{\delta v_{⊥x,i}}{\delta x_{i}}$ also assumes a zero value. Equation (15) presents the simplification of the shear rate tensor acting on a pixel, where $\gamma_{i}$ is the shear rate acting on pixel *i* and $\nabla v_{i}$ is the velocity vector across pixel *i*.

(15) $$\gamma_{i}={\left(\nabla v_{i}\right)}^{T}=\begin{array}{ccc} \frac{\delta v_{⊥x,i}}{\delta x_{i}} & \frac{\delta v_{⊥y,i}}{\delta x_{i}} & \frac{\delta v_{⊥z,i}}{\delta x_{i}}\\ \frac{\delta v_{⊥x,\;i}}{\delta y_{i}} & \frac{\delta v_{⊥y,i}}{\delta y_{i}} & \frac{\delta v_{⊥z,i}}{\delta y_{i}}\\ \frac{\delta v_{⊥x,\;i}}{\delta z_{i}} & \frac{\delta v_{⊥y,i}}{\delta z_{i}} & \frac{\delta v_{⊥z,i}}{\delta z_{i}} \end{array}=\begin{array}{ccc} 0 & 0 & 0\\ \frac{\delta v_{⊥x,\;i}}{\delta y_{i}} & 0 & 0\\ \frac{\delta v_{⊥x,\;i}}{\delta z_{i}} & 0 & 0 \end{array}$$

To obtain values for the nonzero components of the shear rate tensor for each pixel, the velocity gradient was obtained using Equations (16) and (17). All voxels are of equal size and have a square face, where $\delta y_{i}$ is equal to $\delta z_{i}$ , so the spatial difference is denoted as *L*, the length of one voxel.

(16) $$\gamma_{z,i}=\frac{v_{⊥z,i+1}-v_{⊥z,i-1}}{L}$$

(17) $$\gamma_{y,i}=\frac{v_{⊥y,i+1}-v_{⊥y,i-1}}{L}$$

where $\gamma_{z,i}$ and $\gamma_{y,i}$ are the *z* and *y* components of streamwise shear rate across pixel *i*. To map the shear rate distribution, the nonzero components for each pixel in the ROI were computed using a convolution matrix that performed the operations in Equations (16) and (17) on each voxel.

The remaining shear rate components can then be resolved as in Equation (18) to give the overall shear rate acting over the voxel *i* by using the Frobenius norm.

(18) $$\|\gamma_{i}\|=\sqrt{{\left(\frac{\delta v_{xi}}{\delta z_{i}}\right)}^{2}+{\left(\frac{\delta v_{xi}}{\delta y_{i}}\right)}^{2}}$$

#### 2.4.2. DCMDT Data Analysis

In the DCMDT, the shear rates were calculated from the stress tensor shown in Equation (19). The components *σ* define the local normal stress and *τ* the local shear stress in the *xy*-plane, *xz*-plane, and *yz*-plane respectively. Because only the velocity component in the streamwise direction (*x*-direction) is available from the DCM data, the stress tensor can be simplified. The simplification reduces the stress tensor to the local stress on the *yx*-, and *zx*-plane, assuming zero values for all other elements. This facilitates comparison to the experimental data:

(19) $$\tau =\left[\begin{array}{ccc} \sigma_{x} & \tau_{xy} & \tau_{xz}\\ \tau_{yx} & \sigma_{y} & \tau_{yz}\\ \tau_{zx} & \tau_{zy} & \sigma_{z} \end{array}\right]=\left[\begin{array}{ccc} 0 & 0 & 0\\ \tau_{yx} & 0 & 0\\ \tau_{zx} & 0 & 0 \end{array}\right].$$

The remaining shear stress components were condensed into a single value using the Frobenius norm:

(20) $$\|\tau \|=\sqrt{{\left(\tau_{yx}\right)}^{2}+{\left(\tau_{zx}\right)}^{2}}.$$

For simplicity, a Newtonian fluid was used in the computational part. Thus, for the calculation of the shear rate $\dot{\gamma }$ , the following relationship between shear stress, shear rate and fluid velocity was used:

(21) $$\dot{\gamma }=\frac{\tau }{\eta }\;\;\;,$$

where *τ* is the shear stress and *η* the dynamic viscosity of the fluid.

#### 2.4.3. In Vitro and In Silico Comparison Data Analysis

For each combination of parameters, the total sum of squares (*TSS*) between the different velocity data sets was calculated to evaluate the correlation of the experimental and computational data and the difference between the mean and peak measurements inside the DCM:

(22) $$TSS_{j}=\overset{n}{\underset{i=1}{\sum }}{\left(y_{j,i}-x_{j,i}\right)}^{2}\;\;\;,$$

where $y_{j,i}$ and $x_{j,i}$ are the discrete datapoints of a data set *j* which should be compared (i.e., computational data and experimental data). The *TSS* is calculated for each colon section and data set *j* separately.

The main effects of three factors—wave speed, media viscosity and volume—on the response and mean shear rate at the bottom wall during local contractile activity were estimated and visualised using a main effects plot (see Figure 14). Main effects plots (also known as a design of experiment mean plot) are an efficient data visualisation technique that help to identify differences between mean values of experiment parameters and thus depict how individual luminal parameters may influence the shear rate.

## 3. Results and Discussion

### 3.1. Wall Motion

Figure 7, Figure 8, Figure 9, Figure 10 and Figure 11 show the mean displacement (denoted as ‘Wall displ.’) of the mimic intestinal wall beside the consequential velocity profiles of the lumen contents in both the DCM and the DCMDT over the course of a PPW. In both models, the PPW starts at segment 1 (left-hand side), and propagates to segment 10, over the course of 60 s for the slower wave and 35 s for the faster wave. Positive and negative wall displacement represent contraction and relaxation respectively. Figure 7, Figure 8, Figure 9, Figure 10 and Figure 11 demonstrate that the motility pattern of the DCMDT generally corresponded very well with that of the DCM in segments 2, 6 and 10, following an almost identical course of relaxation to −20% occlusion, contraction to 60% occlusion and subsequently a slower relaxation back to the neutral position. This shows that the computational model is suitable to replicate the contractile nature of the DCM walls and can be synchronised to follow the same peristaltic PPW along the colonic axis.

### 3.2. Velocity Profile of the Contents

To verify, the DCMDT can mimic the DCM under a range of environmental conditions and the fluid velocity profiles were compared with those measured in the DCM in all combinations of PPW speed, media viscosity, and luminal fluid volume. In all cases, the DCMDT generated flows of the contents that followed the same pattern as the contents of the DCM. Before a PPW began, the contents were stationary with no measurable velocity. Low fluctuations in velocity between approximately 0.25 and −0.25 cm s^−1^ occurred prior to local wall displacement. Initial relaxation of the walls and contraction of the immediately upstream segment caused positive flows, propelling the contents towards the mimic *hepatic flexure*. Subsequently, contraction of the walls reversed the fluid direction and drove fluid backwards towards the *caecum* at greater velocities. The fluid–structure interactions modelled in the DCMDT were therefore suitable to reproduce the complex series of antegrade propulsion and back mixing observed in the DCM [7,12]. Both models show similarity to the in vivo situation as the velocity of the human ascending colonic contents is also not constant and exhibits periods of rhythmic back and forth motion [49].

Overall, the PPW generated mean fluid velocities in the DCMDT of similar magnitude to that of the DCM. The mean fluid velocities at lower fluid viscosity conditions were slightly noisier than at higher fluid viscosities (for example Figure 7a versus Figure 7b). The DCM produced mean (Figure 7, Figure 9, Figure 10 and Figure 11) and peak (Figure 8) velocities of slightly higher magnitude during the fluctuations above and below the datum outside of the period of local wall contraction. Where small deviations in wall displacement were observed, there was no significant effect on mean velocity of the contents in either the DCM or the DCMDT. More detailed flow phenomena were captured in the DCMDT than the DCM as the experimental data were comparatively low in temporal resolution compared to the DCMDT (2 s versus 0.25 s in this study, respectively) which highlights a clear advantage of using the digital twin. The mean fluid velocities using HIVIS were considerably less noisy than with LOVIS due to enhanced dampening of residual oscillatory motion caused before and after the contractile wave passes.

Figure 7 shows the results obtained when the lumen was filled to 60% capacity and the slower PPW (0.4 cm s^−1^) was applied.

At the lower fill volume of 60%, the slow motility wave (Figure 7) generated particularly similar mean fluid velocities in segment 2, close to the *caecum* as demonstrated by the low *TSS* values of 0.23 and 0.18 for LOVIS and HIVIS fluids respectively. In segment 10, the LOVIS experimental data did not show the strong backflow phenomenon that typically occurred during the contraction phase, which, on the other hand, was evident in the DCMDT.

For the parameter combination shown in Figure 7a (i.e., low fluid volume, low fluid viscosity and slow PPW), the peak fluid velocities that occurred in the experiment and the computation are presented in Figure 8.

The peak velocities fluctuated in a wavelike pattern similar to the mean velocities. Despite the similarities between the DCM and the DCMDT upon visual analysis, *TSS* values were relatively high. This was due to the slight phase offset between the wavelike flow pattern of the DCM and the DCMDT which arose from marginally different initiation times. Next, the fill volume of the lumen was increased to 80% (Figure 9).

When volume was increased to 80%, *TSS* values were <1.4 with no significant deviations between the experimental and computational data. This shows that the simulation is robust at the elevated volume when the slower PPW is applied. At this stage, a limitation of the DCM and its DT is that the ‘neutral’ volume of the lumen is fixed, so when varying the fill volume of fluid inside the lumen below 100%, an air space is present at the top of the lumen. In vivo, the capacity of the ascending colon adapts according to the volume of its contents; the walls of the colon reduce their tone and encase the contents fully, leaving no air gap (unless gas is present as a product of microbial activity). However, the focus of this paper is to demonstrate that the digital twin can reproduce flows inside the DCM under different luminal conditions. Future in silico models of the human ascending colon could better represent the in vivo situation by incorporating this morphological response to the volume of the contents to understand how this may affect the flow of the contents. The fluid volume was then reduced back to 60% and the faster PPW was applied (Figure 10). A faster PPW involved a faster occlusion rate which caused greater mean fluid velocities compared to the slower PPW seen in Figure 7.

The experimental data shown in Figure 10a segment 2 and segment 6 and Figure 10b segment 2 exhibited a slightly higher mean fluid velocity ahead of the wall wave compared to the slower PPW. These elevated positive velocities were also accurately reproduced by the DCMDT in addition to the greater magnitude of backflow velocity. Both models also showed a higher fluid velocity in segment 6 at high fluid viscosity Figure 10b.

Inside the DCM, media viscosity influenced the flow pattern, with a lower viscosity fluid causing more erratic wave-like behaviour. From the statistical analysis in Figure 10a, it can also be seen that the DCMDT data do not fully capture this fluid behaviour in the DCM. This could be attributed to shear rates at the extremes of, or outside of the linear viscoelastic region of the NaCMC solutions, causing the behaviour of the real fluid to deviate from that of the simulated fluid in the DCMDT. A small contribution may also result from small irregularities between the segments in the DCM that are not captured in the DCMDT.

In Figure 11, the faster PPW was maintained but fill volume was increased from 60% to 80%. In this case, there were no significant changes in mean velocity in the DCM that arose from increasing the fill volume from 60% to 80%. The DCMDT performed well to capture this as shown by a relatively low *TSS*.

Generally, mean velocities were slightly higher in the DCM than in the DCMDT.

Comparison of Figure 12 parts (a) and (b) demonstrates the influence of propagating wave on the velocities achieved by the contents of the lumen, which follows intuition that a faster wave produces higher velocities in both the DCM and the DCMDT.

The antegrade velocities were less affected than the retrograde peak during local wall contraction. A lower fill volume increased the degree of retrograde velocity experienced in the DCM, and this was replicated in the DCMDT also. Increasing fluid viscosity in the DCMDT decreased average retrograde velocity during local wall contraction, however, there was no significant effect in the DCM.

#### 3.2.1. Shear Rates

**Figure 13:**
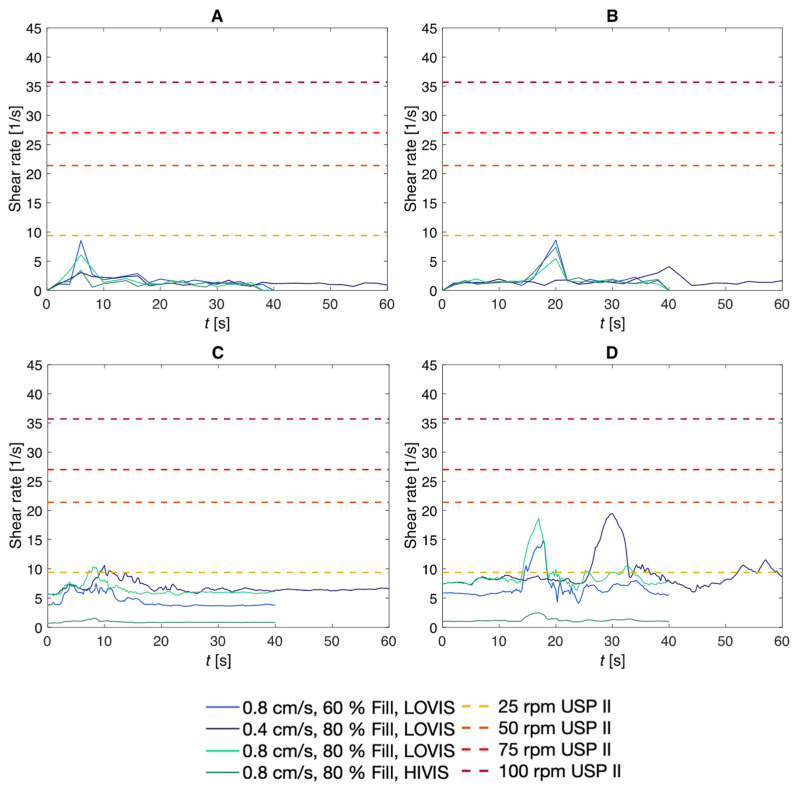
Average shear rates versus maximum shear rates for each parameter combination, where (**A**) represents DCM ‘segment 2’, (**B**) DCM ‘segment 6’, (**C**) DCMDT ‘segment 2’, and (**D**) DCMDT ‘segment 6’. USPII shear rate data was reproduced from [50], Elsevier, 2018.

Figure 13 presents the mean shear rate over time in the same cross section, and the maximum shear rate recorded for each of the same parameter combinations. In the DCM, mean shear rate spiked during local contraction of the walls at approximately 6 s and 20 s for the fast wave in segments 2 and 6 respectively in Figure 13A,B. Subsequently, shear rate dropped sharply, returning to low levels where small fluctuations between 0.01 s^−1^ and 3 s^−1^ were seen for the remainder of the motility wave. For the slow wave, local contractile activity occurred around 6 s and 40 s in segments 2 and 6 respectively, causing a lower, broader peak in average shear rate.

In the digital twin, a similar trend was observed in that there was a peak in average shear rate during a local wall contraction. However, instead of returning to low levels immediately, the average shear rate in segment 2 (Figure 13C) followed the general trend of decreasing post-contraction but periodically peaking to progressively lower shear rates as the subsequent segments contract. This effect was most prominent with the slow wave at a low viscosity and the greater volume of 80%, which also gave rise to the highest mean wall shear rates in segment 6, peaking at 19.48 s^−1^ and in segment 2 at a height of 10.60 s^−1^. In segment 6, shear rates were considerably higher than in segment 2, however, the periodic increases in shear rate following the highest peak arising from local contraction were irregular and less well defined. This suggests that a tablet located close to the *caecum* might experience more frequent peaks in shear rate and may erode faster, according to findings from a recent in silico study which suggested that is not the average shear rate that is important for tablet disintegration in the colon, but individual shear rate peaks that lead to accelerated tablet disintegration [4]. In both segments 2 and 6, shear rates were considerably lower when the lumen contained the higher viscosity fluid, HIVIS. Even though the DCMDT and DCM data show deviations in their course, the order of magnitude of the computational and the experimental data agree well.

The mean bottom wall shear rate in both the DCM and the digital twin were highly variable and time-dependent, in contrast to inside the USPII modelled by Hopgood and Barker [50]. In the USPII model, tablet surface shear rates were approximately constant for a given paddle speed and increased linearly from 9 s^−1^ at 25 rpm to 36 s^−1^ at 100 rpm [50]. At no combination of parameters covered in this study does the shear rate at the wall in the DCM or DCMDT reach that of the USPII at 50 rpm (21.4 s^−1^) or higher. This finding suggests that a constant paddle rotational speed greater than 50 rpm may bear low physiological relevance when studying the dissolution of colon-targeted dosage forms in the USPII. The spatiotemporal dependence of wall shear rate in the DCM is in line with observations in a CFD simulation of the TIM-Automated Gastric Compartment, which is a similar advanced biorelevant in vitro dissolution apparatus modelling the stomach [50].

**Figure 14:**
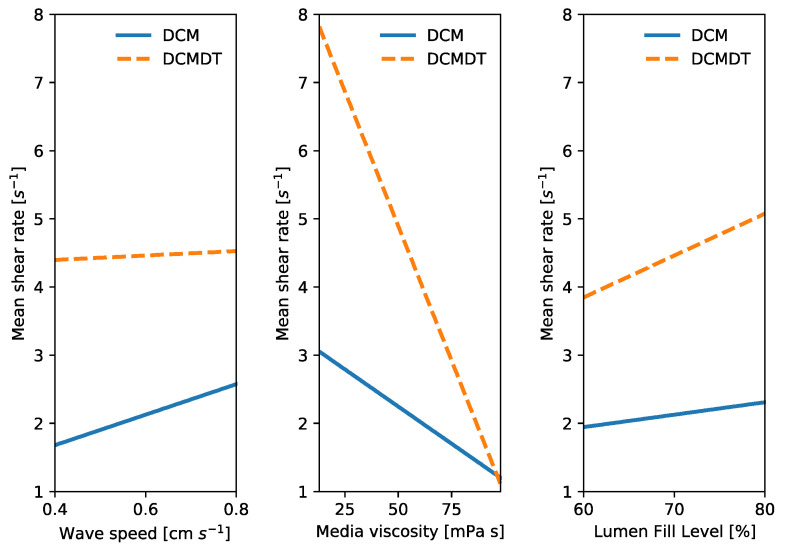
Main effects of wave speed, media viscosity and volume on mean shear rate at the bottom wall during local wall contraction at segment 6 in the DCM and the COM. N = 4 mean data points at each level (low and high).

Clearly, Figure 13 showed that mean wall shear rate in both models had some dependence on the speed of the propagating wave, media viscosity and media volume. The main effects plot in Figure 14 scrutinises this further, giving a clearer idea about the relative significance of these parameters on the mean wall shear rate.

In all plots, the DCMDT is shown to represent the same type of effect as the DCM. Weak positive effects of wave speed and media volume and a strong positive effect of media viscosity on mean shear rate at the bottom wall during local wall contraction were evident. This shows that the DCMDT can model the influences of changes in wave speed, media viscosity, and volume on magnitude of luminal flow velocity. Effects were more pronounced in the DCMDT than the DCM.

Over the parametric range studied in this work, only the effect of media viscosity on mean shear rate was significant (*p* < 0.05) in both models. This demonstrates that media viscosity is a key parameter to consider when designing a biorelevant media for dissolution testing, since shear rate influences dissolution rate. Furthermore, this may mean that colonic disease states that alter media viscosity may divert the intended release profile towards a dose-dump-type scenario or the opposite, insufficient release and therefore administration of therapeutic molecules to the target site in vivo.

Although the main effects of wave speed and media volume on mean shear rates are insignificant at between 0.4–0.8 cm s^−1^ and 60%–80% fill level, the main effects plot suggests that these parameters may demonstrate some influence on shear rate over a broader range of levels. Considering wave speed, a recent study in the DCM showed that wave propagation speed increases the velocity of the contents due to the higher level of kinetic energy imparted to the luminal fluid [12]. Intuitively, this may cause steeper velocity gradients and therefore higher shear rates. Future work should therefore consider a wider range of wave propagation speeds. The range of speeds in this study (0.4–0.8 cm s^−1^) covers fed cyclic antegrade (0.8 ± 0.3 cm s^−1^) and fed short single antegrade (0.5 ± 0.3 cm s^−1^) [13]. However, long single waves have been reported to propagate at (2.0 ± 0.8 cm s^−1^) [13]. Other factors are at play in a motility pattern other than propagation velocity, for example, high amplitude propagating sequences (HAPSs, 0.4 ± 0.1 cm s^−1^ [13], 0.71 (0.29–5.15, solid-state catheter, 0.76 (0.22–6.06, water perfused catheter [51]), 1.11 ± 0.1 cm s^−1^ [52]) which have a similar velocity, exhibit a higher pressure amplitude as a result of higher occlusion rate and/or degree, which is likely to influence shear rate. Also, it is unknown how a retrograde propagating contractile wave affects flow in the DCM or its digital twin. Increasing volume influences the pressure and gravitational forces associated with fluid inside the lumen during a contraction, which is likely to influence shear rates. Future hydrodynamic investigations could explore the effect of orientation of the DCM and DCMDT and the associated influence of gravity on shear rates.

As already mentioned, the size of the DCM segments is fixed so that the membrane does not adjust to the current amount of intestinal content. This feature is also difficult to visualise in practice. However, the DT might offer a feasible way to represent the in vivo environment in a more realistic way by implementing this feature to investigate how this effects shear rates, along with adding in the complexities of gravity by standing the model up so that the *hepatic flexure* is above the *caecum*—as is the case in normal life.

## 4. Conclusions

The alignment of advanced in vitro and in silico models of in vivo systems is a promising approach to begin addressing the gaps in knowledge that currently hamper the progression of drug delivery and disease therapy. This study describes the development of a digital twin of the Dynamic Colon Model, a biorelevant dissolution apparatus representing the human proximal colon. The capabilities of the digital twin were verified using fluid velocity and shear rate data obtained through MRI imaging of the in vitro model. The DCMDT presents an addition to the available toolbox of in silico frameworks to model the fate of orally ingested dosage forms inside the gastrointestinal tract.

In the colon, hydrodynamic parameters such as shear rates are pivotal in the disintegration and dissolution of a solid dosage form, particularly erodible matrices. Both models permit modification of a range of physiologically relevant parameters that describe the colonic environment and influence the hydrodynamic conditions inside the respective mimic lumen. This study investigated the effects that the propagation speed of a contractile wall wave, media viscosity, and media volume have on the mean wall shear rate inside the Dynamic Colon Model. It was found that media viscosity had a significant negative effect on wall shear rate, whilst weak positive effects were seen by propagating wave speed and media volume, which are anticipated to be enhanced at more extreme levels. The digital twin was able to replicate these effects, meaning that it is robust over a range of physiologically relevant parameter combinations and may be useful to model particular disease states and the effect these may have on the delivery of colon-targeted dosage forms.

The findings in this paper indicate that viscosity is important to consider when designing a biorelevant media for dissolution testing of colon-targeted dosage forms. Additionally, constant paddle rotational speed greater than 50 rpm may bear low physiological relevance when studying the dissolution of colon-targeted dosage forms in the USPII dissolution apparatus. However, to consolidate the findings of this study, further work needs to be done that also considers the different motility conditions (i.e., wave speeds, direction of propagation and occlusion degrees) found in the colonic environment.

## Figures and Tables

**Table 1:** MRI scanner parameters.

Parameter	Value
Scan duration [s]	60
TR [ms]	9.21
TE [ms]	7.60
FA [°]	10
FOV [mm^2^]	177 × 200
Recon resolution [mm^2^]	1.1 × 1.1
Slice thickness [mm]	8
SENSE	2.0
No. dynamics	30
Temporal Resolution [s]	2

**Table 2:** Model parameter of the membrane.

Parameter	Value
**SPH**	
Total number of membrane particles (one layer)	2500
Number of membrane particles (DCMDT)	975
Mass of each particle *m*	3.89 × 10^−4^ kg
**LSM**	
Hookean coefficient (bonds) *k_M,b_*	0.1 J m^−2^
Hookean coefficient (position) *k_M,p_*	0.012 J m^−2^
Viscous damping coefficient *k_M,v_*	1.0 × 10^−2^ kg s^−1^
Equilibrium distance *r_0_*	6.283 × 10^−3^ m

**Table 3:** Fluid rheological model parameter.

Fluid	*K* [Pa s^n^]	*n* [-]
Low viscosity fluid (LOVIS)	0.04	0.87
High viscosity fluid (HIVIS)	0.20	0.74

**Table 4:** Model parameter of the fluid.

Parameter	Value
**SPH**	
Number of fluid particles (150 mL/60% filling level)	11,507
Number of fluid particles (200 mL/80% filling level)	18,076
Mass of each fluid particle *m_F,low viscosity_*	1.324 × 10^−5^ kg
Mass of each fluid particle *m_F,high viscosity_*	1.328 × 10^−5^ kg
Density (fluid) *ρ_F,low viscosity_*	1017 kg m^−3^
Density (fluid) *ρ_F,high viscosity_*	1020 kg m^−3^
Dynamic viscosity (fluid) *η_F,low viscosity_*	26 mPa s
Dynamic viscosity (fluid) *η_F,high viscosity_*	85 mPa s

**Table 5:** Fundamental model parameter.

Parameter	Value
**SPH**	
Artificial speed of sound *c*_0_	0.1 m s^−1^
Time-step Δ*t*	5 × 10^−4^ s
Smoothing length, *h*	4.71 × 10^−3^ m
Momentum-Smoothing length, *h_M_*	9.42 × 10^−3^ m

## Data Availability

The data that support the findings of this study and code used for the simulations are freely available on request from the corresponding author.
