# Correction: Schütt et al. Simulating the Hydrodynamic Conditions of the Human Ascending Colon: A Digital Twin of the Dynamic Colon Model. *Pharmaceutics* 2022, *14*, 184

**DOI:** 10.3390/pharmaceutics14071402

**Published:** 2022-07-04

**Authors:** Michael Schütt, Connor O’Farrell, Konstantinos Stamatopoulos, Caroline L. Hoad, Luca Marciani, Sarah Sulaiman, Mark J. H. Simmons, Hannah K. Batchelor, Alessio Alexiadis

**Affiliations:** 1 School of Chemical Engineering, University of Birmingham, Edgbaston, Birmingham B15 2TT, UK; konstantinos.x.stamatopoulos@gsk.com (K.S.); m.j.simmons@bham.ac.uk (M.J.H.S.); 2 Biopharmaceutics, Pharmaceutical Development, PDS, MST, RD Platform Technology & Science, GSK, David Jack Centre, Park Road, Ware, Hertfordshire SG12 0DP, UK; 3 Nottingham Digestive Diseases Centre and National Institute for Health Research (NIHR) Nottingham Biomedical Research Centre, Nottingham University Hospitals NHS Trust and University of Nottingham, Nottingham NG7 2UK, UK; caroline.l.hoad@nottingham.ac.uk (C.L.H.); luca.marciani@nottingham.ac.uk (L.M.); sarah.sulaiman@nottingham.ac.uk (S.S.); 4 Sir Peter Mansfield Imaging Centre, School of Physics and Astronomy, University of Nottingham, Nottingham NG7 2RD, UK; 5 Strathclyde Institute of Pharmacy and Biomedical Sciences, University of Strathclyde, 161 Cathedral Street, Glasgow G4 0RE, UK; hannah.batchelor@strath.ac.uk

In the original publication [1], there was a mistake in ***Figure 6*** when published. The experimental data points in the upper diagram were missing. The corrected ***Figure 6*** appears below. The authors apologize for any inconvenience caused and state that the scientific conclusions are unaffected. This correction was approved by the Academic Editor. The original publication has also been updated.

## Figures and Tables

**Figure 6:**
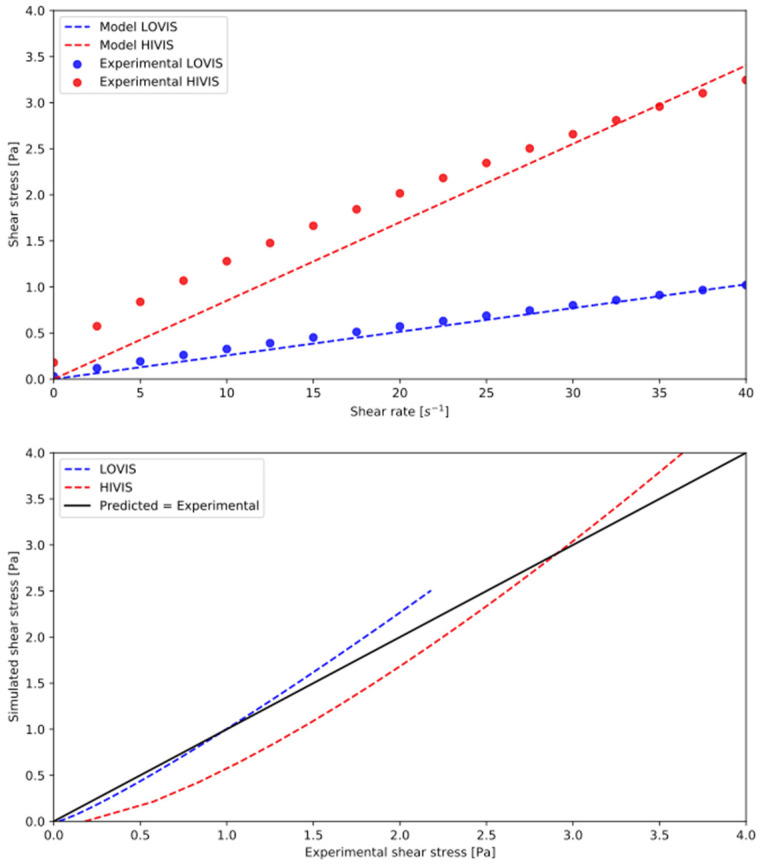
Rheological behavior of LOVIS and HIVIS fluids in the DCM and the simulated counterparts in silico. Rheological measurements were carried out at 25 °C.
